# Empirically Derived Whole-Diet Patterns and Multidimensional Adiposity in Emerging Adulthood: A Cross-Sectional Study of Geographically Diverse Students at a UK University

**DOI:** 10.3390/nu18203320

**Published:** 2026-10-10

**Authors:** Antonio Peña-Fernández, Tomás Cámara-Pastor, M. Ángeles Peña Fernández

**Affiliations:** 1 Area of Legal and Forensic Medicine, Department of Surgery, Medical and Social Sciences, Faculty of Medicine and Health Sciences, University of Alcalá, Ctra. Madrid-Barcelona, Km. 33.6, 28871 Alcalá de Henares, Spain; 2 Leicester School of Allied Health Sciences, De Montfort University, Leicester LE1 9BH, UK; 3 Scientific Computation Research Institute (SCRIUR), University of La Rioja, 26006 Logroño, Spain; 4 Department of Biomedical Sciences, Faculty of Pharmacy, University of Alcalá, Crta. Madrid-Barcelona Km. 33.6, 28871 Alcalá de Henares, Spain

**Keywords:** dietary patterns, adiposity, body composition, body mass index, visceral fat, emerging adulthood, university students, principal component analysis, young adults, United Kingdom

## Abstract

**Background**: Emerging adulthood is a period in which dietary behaviours may become established before their longer-term effects on adiposity are fully expressed. Evidence linking empirically derived whole-diet patterns with multiple adiposity phenotypes remains limited in narrow-age university populations. This study examined associations between three previously derived dietary pattern scores and adiposity/body composition in geographically diverse young adults attending an English university. **Methods**: This historical cross-sectional analysis included 111 students aged 18–22 years assessed during 2016, within the 2015/2016 academic year, at De Montfort University, Leicester. Habitual diet was assessed using an adapted EPIC-Norfolk food frequency questionnaire. Three standardised dietary pattern scores—Plant-Rich, Animal-Source Foods and Energy-Dense/Discretionary—were evaluated in relation to BMI, body fat percentage and a bioelectrical impedance-derived visceral fat index. The prespecified primary multivariable models evaluated dietary pattern–adiposity associations conditional on age, sex, geographical background and log-transformed total energy intake. HC3 robust standard errors were used, with Benjamini–Hochberg false discovery rate correction across the nine prespecified primary associations. **Results**: Mean age was 20.4 ± 1.1 years and 70.3% of participants were female. Overall, 33.3% had BMI ≥ 25 kg/m^2^, whereas visceral fat index values ranged from 1 to 9. None of the nine Model 1 dietary pattern–adiposity associations was nominally statistically significant before adjustment for total energy intake, and addition of energy intake did not uniformly attenuate the coefficients. None of the prespecified primary Model 2 associations survived false discovery rate correction. The most pronounced primary estimate was the inverse association between Animal-Source Foods and visceral fat index (standardised β = −0.172, 95% CI −0.358 to 0.014; *p* = 0.069; *q* = 0.481). Secondary, mutually adjusted, transformed-outcome, geographical background, physical activity-adjusted and influence analyses did not materially alter the overall interpretation. **Conclusions**: After conditioning on total energy intake, no robust independent association was detected between the three empirical dietary pattern scores and contemporary adiposity in this cohort. The findings therefore suggest limited detectable independent relevance of dietary pattern composition for contemporary adiposity, although smaller associations cannot be excluded with the available precision. The results are compatible with overall energy intake capturing adiposity-related variation overlapping with dietary pattern composition but do not establish that energy intake is a stronger causal determinant.

## 1. Introduction

Emerging adulthood represents a distinct life course period during which increasing autonomy over food purchasing, meal preparation, eating occasions and other lifestyle behaviours coincides with major changes in residence, social networks, academic demands and financial circumstances [1,2]. Entry into higher education can intensify these transitions and has been associated with changes in dietary intake, physical activity and body weight [3,4]. The university years are therefore relevant not only for characterising current nutritional status but also as a potential window during which behaviours contributing to later cardiometabolic trajectories become established.

Dietary exposures are frequently investigated at the level of individual foods or nutrients. However, foods are consumed in correlated combinations, and the health relevance of one dietary component may depend partly on the broader dietary context in which it occurs. Dietary pattern analysis provides a complementary approach by characterising the overall structure of habitual food consumption [5,6]. Empirical methods such as principal component analysis (PCA) are particularly useful for identifying combinations of foods that co-occur within a specific population, while continuous pattern scores quantify the degree to which each participant expresses those dietary dimensions.

University populations show substantial heterogeneity in such patterns. In a multicentre study of 1448 students attending five UK universities, Sprake et al. identified vegetarian, snacking, health-conscious and convenience/red meat/alcohol patterns, with pattern adherence varying across demographic and behavioural characteristics [7]. Among 1323 US undergraduates, Blondin et al. derived Prudent, Western and Alcohol patterns and observed favourable cross-sectional associations between greater Prudent pattern adherence and BMI/body fat outcomes [8]. In Chinese medical university students, empirically derived Western, meat and vegetable/fruit patterns were likewise evaluated against a broader body composition phenotype including BMI, body fat percentage and visceral fat index [9]. These studies demonstrate the potential value of dietary pattern approaches in young adults, but also emphasise that empirically derived patterns are population-specific and should not be assumed to correspond directly to generic labels such as “healthy”, “Western” or “plant-based”.

Cross-sectional associations in very young adults may not fully capture longer-term adiposity trajectories. Longitudinal evidence indicates that dietary behaviours established in early adulthood can precede subsequent differences in body weight, adiposity and cardiometabolic outcomes [10,11,12]. At the same time, prospective evidence concerning plant-based dietary indices indicates that associations with adiposity depend substantially on food quality, with healthful and less-healthful plant-based patterns showing different relationships [13,14]. Thus, failure to detect a strong contemporaneous diet–adiposity association at approximately 20 years of age does not imply absence of later dietary relevance, and an empirically derived “Plant-rich” component should not be assumed to represent the same nutritional exposure as a healthful plant-based dietary index.

Adiposity itself is multidimensional. BMI is widely used for population surveillance, but does not distinguish fat mass from lean tissue or describe fat distribution. Body fat percentage and measures intended to approximate abdominal or visceral adiposity may therefore provide complementary information, particularly in young populations where BMI-defined excess weight and body composition may not correspond closely. At the same time, bioelectrical impedance-derived visceral fat indices remain indirect proxies and should not be interpreted as equivalent to anatomical visceral adipose tissue quantified by computed tomography or magnetic resonance imaging.

Importantly, the present study examines dietary and body composition data collected from a university cohort assessed during the 2015/2016 academic year, approximately a decade before the present analysis. The value of this dataset lies in the availability of detailed, contemporaneously collected dietary, anthropometric and body composition data in a narrowly defined emerging-adult population assessed under a common protocol. Accordingly, the aim is not to estimate current dietary or adiposity prevalence among UK university students, but to investigate within-cohort relationships between dietary patterns and multidimensional adiposity at the time of assessment. The historical nature of the cohort also permits interpretation against contemporaneous population surveillance and provides a well-characterised reference point for future longitudinal or repeated investigations.

An additional consideration is the role of total energy intake in the interpretation of dietary pattern associations. In nutritional epidemiology, adjustment for total energy intake is not merely a statistical refinement; it changes the contrast being estimated by separating variation related to overall consumption from that related to dietary composition, and its interpretation therefore depends on the underlying research question and causal framework [15,16,17]. Accordingly, in the present study, the fully adjusted primary model was specified to evaluate whether the empirical dietary pattern scores were associated with adiposity conditional on total energy intake, rather than to estimate the total effect of consuming more food represented by a given pattern.

Against this background, we investigated whether three empirically derived whole-diet patterns—Plant-Rich, Animal-Source Foods and Energy-Dense/Discretionary—were associated with BMI, body fat percentage and BIA-derived visceral fat index in young adults aged 18–22 years. Pattern scores were derived independently of the adiposity outcomes and were analysed using a prespecified inferential framework incorporating measured anthropometry, multiple adiposity phenotypes, HC3 robust standard errors and false discovery rate control. We hypothesised that the three dietary dimensions could demonstrate differential relationships with adiposity/body composition without making directional assumptions based solely on their descriptive PCA labels.

## 2. Materials and Methods

### 2.1. Study Design, Setting and Participants

This historical cross-sectional study examined dietary, anthropometric and body composition data collected from the university component of a wider nutritional and biomonitoring investigation initiated during the 2015/2016 academic year at De Montfort University (DMU), Leicester, UK [18]. The present analysis was restricted to participants with the quality-controlled dietary, anthropometric and body composition information required to address the dietary pattern–adiposity research question.

Recruitment was undertaken over several months through complementary routes across the university. Students were informed about the study through short presentations delivered during scheduled teaching sessions, announcements within Blackboard virtual learning environments, posters and other study materials displayed on campus and dissemination through student or course representatives [18]. Because these strategies overlapped and individual students could encounter recruitment information through more than one route, a reliable denominator for all students exposed to recruitment materials was unavailable; consequently, a formal response rate was not calculated.

Students expressing interest received written information about the study and were offered flexible face-to-face appointments. A £5 voucher was provided under an approved protocol amendment in recognition of the time required for participation. Participation was voluntary, and written informed consent was obtained before any study procedure was undertaken.

Study visits took place in the Biomedical and Environmental Health Research Laboratory, Leicester School of Allied Health Sciences, DMU. Eligibility for the university component comprised enrolment as a DMU student, age 18–22 years and provision of informed consent. For inclusion in the present nutritional analysis, dietary information also had to be sufficiently complete and internally interpretable for quality-controlled processing. The final analytical cohort comprised 111 participants. No values required for the prespecified primary analyses were missing; consequently, all nine primary models included the complete analytical sample (*N* = 111).

Geographical background was derived from recorded continent of birth and classified as Africa, Asia, Europe or Other. It was used as a broad geographical descriptor and adjustment variable and was not regarded as a measure or proxy for genetic ancestry, ethnicity, cultural identity, religion, socioeconomic circumstances, migration experience or acculturation. The Other category comprised two participants. These participants were retained in the primary analysis and descriptive summaries. Exclusion of this sparse category was examined separately as a sensitivity analysis. Additional exploratory sensitivity analyses examined whether dietary pattern associations differed across the three main geographical background categories.

The study was initiated during the 2015/2016 academic year. Study planning and preparation of participant-facing materials began in late 2015 as part of the ethics application process. Formal ethical approval was subsequently granted by Chair’s Action by the Faculty Research Ethics Committee, Faculty of Health and Life Sciences, De Montfort University (Ref. 1674; 11 January 2016). Subsequent protocol amendments were approved through the institutional ethics process. Participant recruitment, informed consent and the study procedures contributing data to the present analysis were undertaken following ethical approval.

### 2.2. Anthropometric and Body Composition Assessment

Age and sex were recorded for each participant. Standing height was measured during the study appointment by a registered nurse working within the UK National Health Service. Body weight and body composition estimates were obtained using a Tanita SC-330-S body composition analyser (Tanita, Tokyo, Japan), based on bioelectrical impedance analysis (BIA) [19,20].

Participants received standardised instructions before their appointment to minimise short-term variation in bioimpedance measurements. They were asked to refrain from intake of food, alcoholic beverages, coffee and other caffeinated beverages, smoking and physical exercise for at least 8 h before assessment; water was permitted during this period. These conditions were intended to reduce measurement variability related to recent dietary intake, hydration-related exposures and exercise [19].

The analyser provided body weight and estimates of body fat percentage, total body water, muscle mass, bone mass, visceral fat rating/index and basal metabolic rate (BMR). BMI was independently recalculated from measured height and weight as follows:

BMI (kg/m^2^) = body weight (kg)/height^2^ (m^2^).

For descriptive analyses, BMI was categorised using conventional adult thresholds: underweight, <18.5 kg/m^2^; healthy weight, 18.5–24.9 kg/m^2^; overweight, 25.0–29.9 kg/m^2^; and obese, ≥30.0 kg/m^2^.

The Tanita visceral fat output is an instrument-derived index rather than a direct anatomical measurement of visceral adipose tissue. The manufacturer’s scale identifies ratings of 1–12 as being within its reference range and ratings ≥13 as elevated [20]. This classification was used only for descriptive context and not as a clinical diagnosis. In the present cohort, observed visceral fat values ranged from 1 to 9; consequently, dichotomisation at the manufacturer’s elevated threshold would have produced no exposed group. The visceral fat index was therefore retained as a continuous primary outcome. More generally, BIA-based abdominal/visceral measures should be regarded as indirect proxies rather than interchangeable with magnetic resonance imaging or computed tomography quantification [21]. No criterion method validation against DXA, MRI or CT was available within the present cohort; accordingly, the BIA-derived measures were interpreted as device-derived body composition estimates rather than criterion measurements.

Two additional adiposity measures were derived for secondary analyses. Fat mass was calculated as follows:

Fat mass (kg) = body weight (kg) × body fat (%)/100.

Fat mass index (FMI) was calculated as follows:

FMI (kg/m^2^) = fat mass (kg)/height^2^ (m^2^).

BMR was retained for descriptive purposes only.

### 2.3. Dietary Assessment and Data Quality Control

Habitual dietary intake was assessed using an expanded semi-quantitative food frequency questionnaire (FFQ) developed for the parent study. Its core food frequency structure was based on an adapted version of the EPIC-Norfolk FFQ, version 6 (CAMB/PQ/6/1205), whose underlying nutritional methodology has been described previously [22]. Questionnaire development was also informed by a semi-quantitative FFQ previously used by the research group in young adults, which included 110 food items, incorporated small, medium and large portion size categories and had been evaluated against repeated 24 h dietary recalls [23]. The repeated 24 h dietary recalls referred to the evaluation of the earlier questionnaire that informed instrument development; they were not collected concurrently in the present cohort. For the present study, the instrument was substantially expanded to improve capture of the foods and dietary behaviours relevant to a geographically diverse university population in Leicester. The expanded FFQ used in the present study was not independently revalidated against a reference dietary assessment method within this sample.

The administered questionnaire contained more than 130 food and beverage items and assessed usual consumption over approximately the preceding four weeks. Participants reported consumption using predefined frequency categories ranging from no or infrequent monthly intake to multiple occasions per day. Portion size was recorded relative to food-specific medium serving sizes using small, medium and large categories. The expanded food list covered a broad range of breads and cereals, vegetables, pulses and grains, fruit, meat and meat products, dairy products, fish and seafood, mixed dishes, beverages and other commonly consumed foods. Additional questions captured selected dietary and lifestyle characteristics.

Additional or modified food entries broadened coverage of foods consumed within the study setting and were informed by established UK food composition resources, including *McCance and Widdowson’s The Composition of Foods* [24]. These adaptations were intended to improve dietary capture within the heterogeneous student population rather than to assign foods to participants according to geographical background or to infer culturally specific dietary behaviour.

Completed FFQs were processed using Nutritics^®^ Research Edition, version 5.7 (Nutritics Ltd., Dublin, Ireland). Dietary information was subsequently reconstructed and quality-controlled at the participant level from the original source database. The source gram variable represented weekly intake and was converted once to daily intake by division by seven. Raw and derived variables were retained separately to maintain data provenance.

Food records were standardised and aggregated into nutritionally coherent analytical groups using an audited mapping framework. Structural zeros were retained for participant–food group combinations absent from the positive-intake source records. The resulting participant-level food group matrix constituted the basis from which the pre-established dietary pattern scores used as exposures in the present study were derived.

### 2.4. Dietary Pattern Exposures

The present analysis used three empirical whole-diet pattern scores—Plant-Rich, Animal-Source Foods and Energy-Dense/Discretionary—that were derived before examination of their associations with adiposity. Pattern derivation used 15 predefined analytical food groups: alcoholic beverages; cereals/grains; dairy/alternatives; discretionary foods; eggs; fats/oils; fish/seafood; fruit; nuts/seeds; poultry; pulses; red/processed meat; soft/energy/sports drinks; tea/coffee; and vegetables.

Because food group distributions contained structural zeros and substantial right-skewness, daily intakes were transformed as ln[1 + intake (g/day)] and standardised before PCA. Component retention considered parallel analysis evidence, scree/eigenvalue structure, loading patterns, communalities, nutritional interpretability and bootstrap stability. The retained three-component solution was subjected to orthogonal varimax rotation with Kaiser normalisation, and participant scores were calculated from the rotated solution and standardised to mean 0 and SD 1. The three components jointly explained 46.25% of food group variance.

The Plant-Rich component was principally characterised by positive loadings for pulses, nuts/seeds, vegetables, cereals/grains and fruit, with a smaller positive loading for fish/seafood. The Animal-Source Foods component was defined principally by red/processed meat, poultry, fish/seafood and eggs, with dairy/alternatives also contributing. The Energy-Dense/Discretionary component was characterised by fats/oils, discretionary foods, soft/energy/sports drinks, alcoholic beverages, cereals/grains and dairy/alternatives. Principal food group loadings and variance explained are provided in Table S1.

Importantly, pattern derivation and participant scoring were completed independently of the adiposity analyses. The three pattern scores were therefore carried forward as pre-established nutritional exposures: the PCA was not re-run, component retention was not reconsidered and pattern labels or participant scores were not modified after inspection of the adiposity outcomes. Pattern labels are descriptive summaries of correlated food consumption within this cohort rather than prespecified indices of dietary healthfulness.

### 2.5. Outcomes and Covariates

Three primary adiposity outcomes were prespecified: BMI, body fat percentage and visceral fat index. Secondary outcomes were body weight and muscle mass. Body water percentage was considered complementary. Fat mass and FMI were derived secondary outcomes, whereas BMR was descriptive only.

The core covariates were age, sex and geographical background. Total energy intake was included in the primary fully adjusted model to distinguish pattern composition from variation associated principally with greater or lower overall dietary intake. Because energy adjustment changes the contrast being estimated and its interpretation depends on the underlying research question, Model 2 was specified to estimate the association between dietary pattern composition and adiposity conditional on total energy intake [15,16,25]. Age and total energy intake were modelled continuously; total energy intake was natural-log transformed before standardisation. Height was additionally included in models for weight, muscle mass and fat mass because these absolute mass outcomes are strongly dependent on body size. BMI was not included as a covariate in models of body fat percentage or visceral fat index because it represents an overlapping adiposity phenotype rather than an independent confounder for the present research question.

Physical activity was assessed using the Recent Physical Activity Questionnaire (RPAQ) [26], with participant-level variables available for sedentary, light, moderate and vigorous activity. For the present analysis, moderate and vigorous activity were combined to derive an MVPA measure, which was standardised before analysis. Physical activity was not included in the prespecified primary models, but MVPA was incorporated as an additional covariate in a sensitivity analysis to assess the robustness of the primary dietary pattern–adiposity associations.

### 2.6. Primary Statistical Analysis

All definitive analyses were conducted in R version 4.4.2 (R Foundation for Statistical Computing, Vienna, Austria) [27]. Continuous variables are described using mean ± standard deviation and median [interquartile range] where appropriate; categorical variables are reported as *n* (%).

The analytical sample size was fixed by the number of eligible participants with complete data available from the parent study; no outcome-specific prospective sample size calculation was performed. A fixed-sample sensitivity analysis indicated that, with *N* = 111, the minimum detectable incremental effect for a single dietary pattern term at 80% power and a nominal two-sided *α* = 0.05 was approximately *f*^2^ = 0.076 (partial *R*^2^ = 0.071), before multiplicity correction. Inference therefore emphasised effect estimates and 95% confidence intervals together with the prespecified false discovery rate correction.

The three dietary pattern scores were already standardised and were used without further transformation. Continuous outcomes were standardised before regression, allowing dietary pattern coefficients to be interpreted as standardised β estimates representing the difference in outcome SD associated with a 1-SD increment in pattern score.

For each combination of dietary pattern and primary outcome, two multivariable linear models were fitted: Model 1 included the dietary pattern score, standardised age, sex and geographical background. Model 2, designated a priori as the primary model, additionally included standardised log-transformed total energy intake. Thus,

Model 1: outcome = pattern score + age + sex + geographical background.

Model 2: Model 1 + log(total energy intake).

Heteroscedasticity-consistent HC3 robust standard errors were used for primary linear model inference because they provide improved finite-sample protection against heteroscedastic residual variance [28]. Effect estimates are reported with 95% confidence intervals and two-sided *p*-values.

The confirmatory multiplicity family comprised exactly nine Model 2 associations: three dietary pattern scores × three primary adiposity outcomes. Benjamini–Hochberg false discovery rate (BH-FDR) correction was applied jointly to these nine Model 2 *p*-values [29]. Results were interpreted principally from effect estimates, confidence intervals and BH-FDR-adjusted *q*-values rather than isolated nominal *p*-values.

### 2.7. Secondary, Sensitivity, Diagnostic and Exploratory Analyses

Secondary/complementary linear models used the same Model 1 and Model 2 covariate structures. Height was additionally included for weight, muscle mass and fat mass. Model 2 *p*-values for the 15 secondary/complementary pattern–outcome associations were subjected to a separate BH-FDR correction and were not combined with the nine-test primary family.

Several prespecified analyses evaluated robustness. First, all three dietary pattern scores were entered simultaneously into the fully adjusted Model 2 to assess the robustness of pattern-specific estimates when all identified dietary dimensions were represented within the same model. Second, visceral fat index was transformed using log1p and re-standardised to assess sensitivity to its restricted and right-skewed distribution. Third, primary analyses were repeated after excluding the two participants classified in the Other geographical background category.

To further examine potential heterogeneity across geographical backgrounds, exploratory effect modification analyses excluded the two participants classified as Other and refitted each of the nine primary Model 2 regressions with dietary pattern score × geographical background interaction terms. The same outcome and dietary pattern standardisation and HC3 robust inference used in the primary analyses were retained. Interaction terms were evaluated jointly using omnibus robust Wald F tests, and BH-FDR correction was applied separately across the nine interaction tests.

As an additional sensitivity analysis, the nine primary Model 2 associations were refitted, with standardised MVPA included as an additional covariate. The same exposure and outcome standardisation and HC3 robust inference used in the primary analyses were retained, and BH-FDR correction was applied separately across the nine dietary pattern coefficients from this sensitivity analysis. This analysis did not alter the prespecified primary model specification.

Diagnostics were evaluated for each of the nine primary Model 2 regressions using conventional least-squares fits. These comprised residual assessment, the Breusch–Pagan test for heteroscedasticity, design matrix variance inflation factors (VIFs), leverage, Cook’s distance, studentised residuals and the DFBETA corresponding to the dietary pattern exposure. Observations meeting prespecified Cook’s distance (>4/*n*), leverage (>2*p*/*n*) or absolute studentised residual (>3) criteria were removed only within influence refit sensitivity analyses; these diagnostic refits did not replace the primary models or generate automatic exclusions from the main dataset.

Finally, BMI ≥ 25 kg/m^2^ versus <25 kg/m^2^ was evaluated as an exploratory binary outcome using one dietary pattern score at a time in logistic regression models adjusted for age, sex, geographical background and log-transformed total energy intake. Model convergence and extreme fitted probabilities were assessed. BH-FDR correction was applied separately across these three exploratory tests.

The study is reported in accordance with the principles of the STROBE statement for observational cross-sectional studies [30].

### 2.8. Reproducibility and Generative AI

The statistical workflow was specified before preparation of the manuscript, and the analyses reported here derive from a final reproducible R workflow with archived model outputs, diagnostic results and plotting datasets. The dietary pattern scores were treated as fixed exposures and were not re-estimated in response to the adiposity results.

During preparation of the manuscript, ChatGPT (OpenAI; GPT-5 family) was used to assist with manuscript structuring, language drafting and refinement and graphical presentation. It was not used to generate or modify study data, perform the definitive statistical analyses or independently determine the scientific interpretation. All AI-assisted content was critically reviewed, checked against the analytical outputs and edited by the authors, who take responsibility for the final manuscript.

## 3. Results

### 3.1. Characteristics and Adiposity Phenotype of the Study Population

The analytical cohort comprised 111 young adults aged 18–22 years. Mean age was 20.4 ± 1.1 years, and 78 participants (70.3%) were female and 33 (29.7%) were male. Geographical background was classified as Africa for 41 participants (36.9%), Asia for 41 (36.9%), Europe for 27 (24.3%) and Other for 2 (1.8%) (Table 1).

Mean body weight was 66.4 ± 13.5 kg and mean BMI was 23.69 ± 4.09 kg/m^2^. Ten participants (9.0%) were classified as underweight, 64 (57.7%) as healthy weight, 28 (25.2%) as overweight and 9 (8.1%) as having obesity. Therefore, 37/111 participants (33.3%) had BMI ≥ 25 kg/m^2^ overall.

Mean body fat percentage was 25.7 ± 10.4%, while mean visceral fat index was 2.7 ± 2.0. Visceral fat values ranged from 1 to 9, with a median of 2 [IQR 1–4]; thus, no participant reached the manufacturer’s descriptive elevated rating threshold of ≥13. Mean muscle mass was 46.4 ± 8.8 kg and mean body water was 53.0 ± 6.7%. Mean derived fat mass and FMI were 17.7 ± 9.5 kg and 6.39 ± 3.48 kg/m^2^, respectively (Table 1).

### 3.2. Primary Associations Between Dietary Patterns and Adiposity

**Figure 1:**
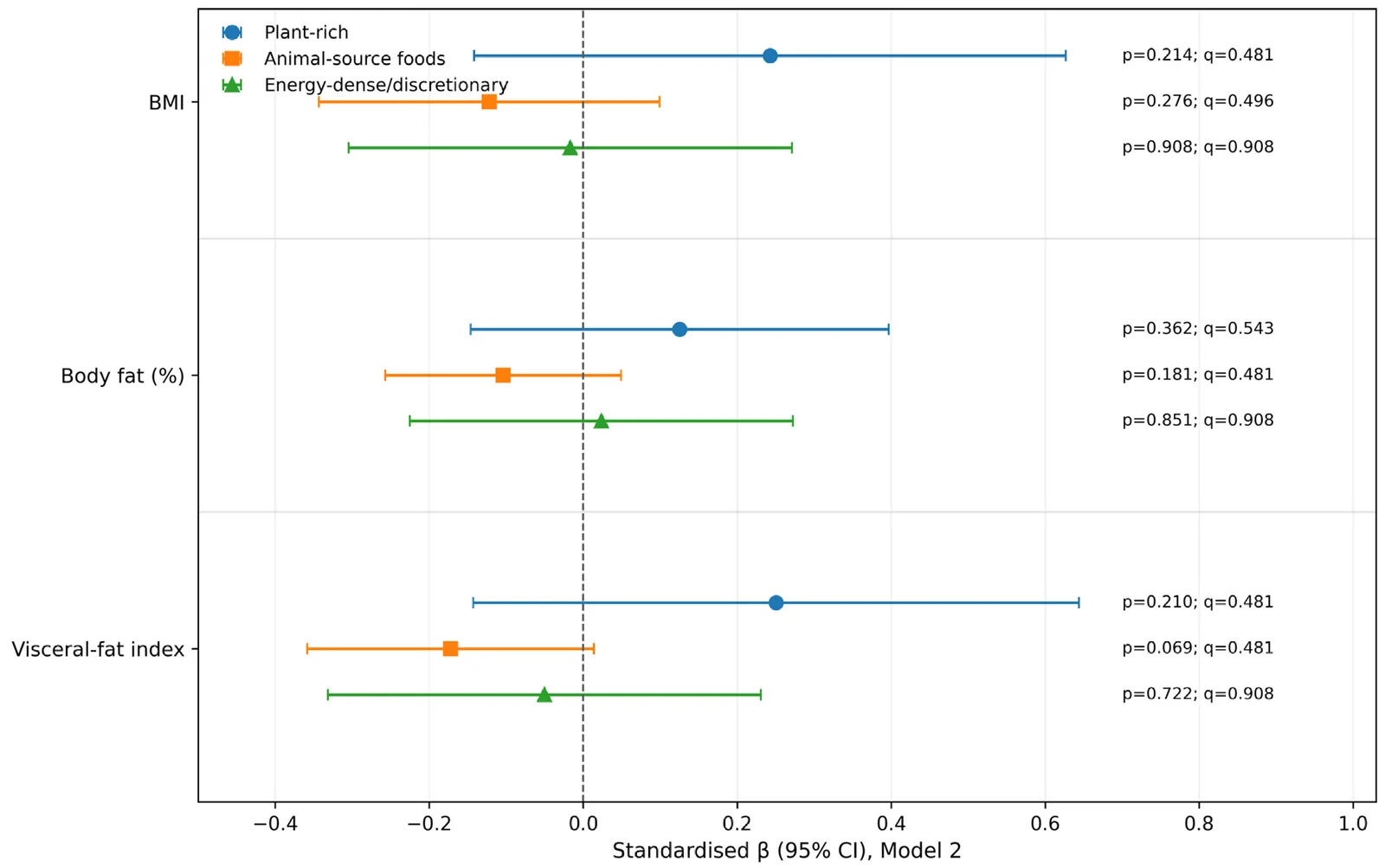
Associations of the three pre-established dietary pattern scores with BMI, body fat percentage and visceral fat index in the primary multivariable model (Model 2). Points represent standardised β coefficients and horizontal bars represent 95% confidence intervals. Models were adjusted for age, sex, geographical background and log-transformed total energy intake, with HC3 robust standard errors used for inference. The dashed vertical line indicates the null value (β = 0). Benjamini–Hochberg false discovery rate correction was applied across the nine prespecified primary associations. BH-FDR, Benjamini–Hochberg false discovery rate; BMI, body mass index; CI, confidence interval.

The nine prespecified primary associations are presented in Table 2 and Figure 1. None met the BH-FDR threshold of *q* < 0.05.

For the Plant-Rich pattern, the fully adjusted Model 2 estimates were positive but imprecise for all three primary outcomes: BMI (β = 0.242, 95% CI −0.142 to 0.627; *p* = 0.214; *q* = 0.481), body fat percentage (β = 0.125, 95% CI −0.146 to 0.397; *p* = 0.362; *q* = 0.543) and visceral fat index (β = 0.250, 95% CI −0.143 to 0.644; *p* = 0.210; *q* = 0.481).

Animal-Source Foods showed negative point estimates across BMI, body fat percentage and visceral fat index. Associations were β = −0.122 (95% CI −0.343 to 0.099; *p* = 0.276; *q* = 0.496) for BMI and β = −0.104 (95% CI −0.257 to 0.049; *p* = 0.181; *q* = 0.481) for body fat percentage. The most pronounced primary estimate was observed for visceral fat index, with β = −0.172 (95% CI −0.358 to 0.014; *p* = 0.069; *q* = 0.481); however, its confidence interval included the null and the association did not meet either the nominal or the BH-FDR-adjusted threshold for statistical significance.

The Energy-Dense/Discretionary pattern showed estimates close to zero for BMI (β = −0.017, 95% CI −0.305 to 0.271; *p* = 0.908; *q* = 0.908), body fat percentage (β = 0.024, 95% CI −0.225 to 0.272; *p* = 0.851; *q* = 0.908) and visceral fat index (β = −0.050, 95% CI −0.332 to 0.231; *p* = 0.722; *q* = 0.908).

None of the nine Model 1 dietary pattern–adiposity associations was nominally statistically significant before adjustment for total energy intake. The smallest Model 1 *p*-values were observed for Plant-Rich score with body fat percentage (β = 0.149, 95% CI −0.019 to 0.317; *p* = 0.081) and Animal-Source Foods with visceral fat index (β = −0.159, 95% CI −0.345 to 0.027; *p* = 0.092). Addition of total energy intake in Model 2 did not uniformly attenuate the dietary pattern coefficients; estimates changed in magnitude in both directions, and no Model 2 association met the prespecified BH-FDR threshold (Table 2). The primary result was therefore an absence of statistically robust contemporaneous associations across the predefined nine-test family, rather than evidence that adjustment for total energy intake eliminated previously significant associations.

### 3.3. Adiposity Phenotype in Descriptive UK Context

**Figure 2:**
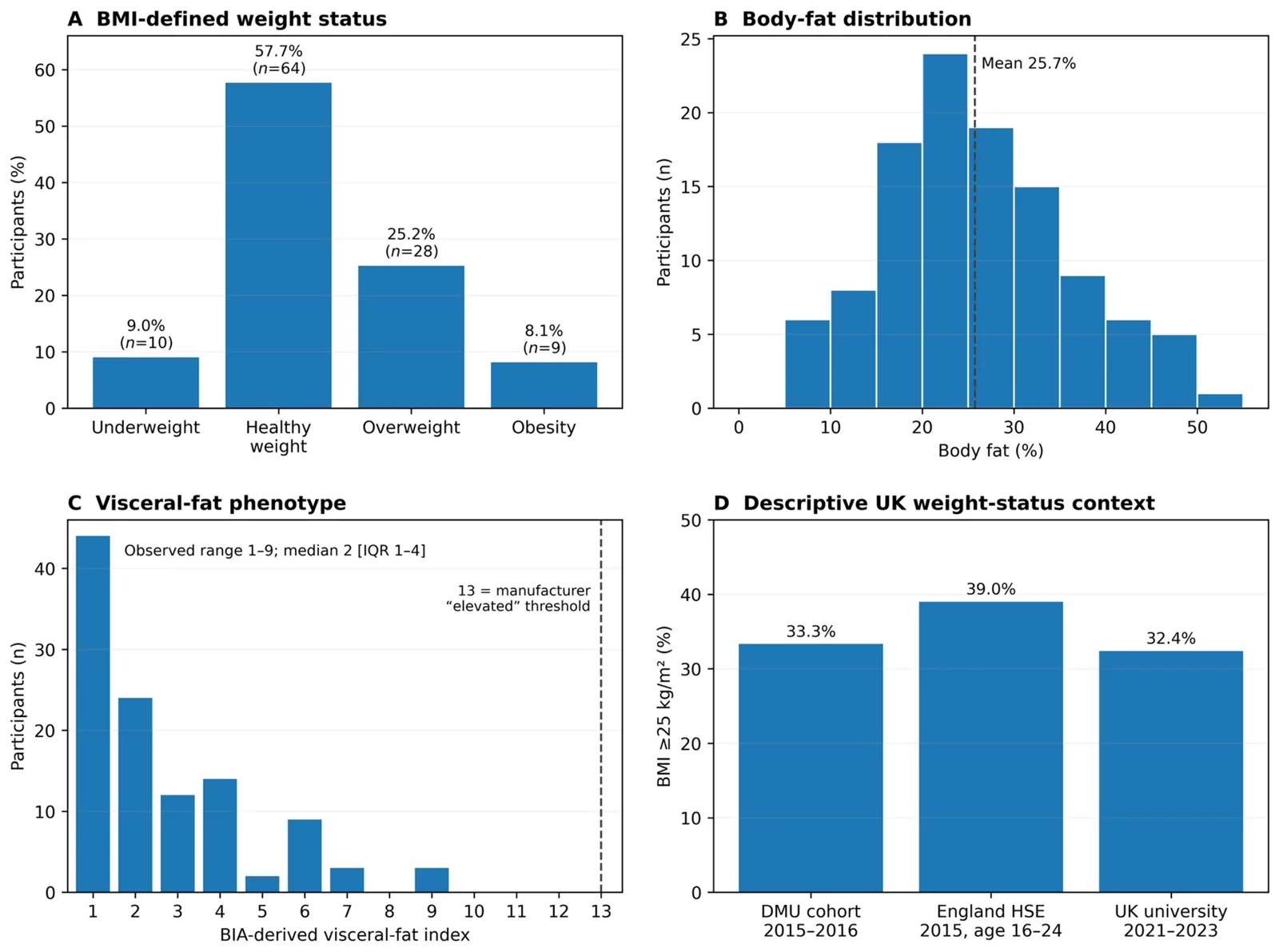
Adiposity phenotype and descriptive weight status context of the DMU young-adult cohort. (**A**) Distribution of participants across conventional adult BMI categories. (**B**) Distribution of body fat percentage in the *N* = 111 cohort; the dashed line marks the cohort mean. (**C**) Distribution of the BIA-derived visceral fat index; observed values ranged from 1 to 9, with a median of 2 [IQR 1–4]. The dashed line at 13 indicates the manufacturer’s threshold for an elevated visceral fat rating [20]. (**D**) Descriptive comparison of the prevalence of BMI ≥ 25 kg/m^2^ in the DMU cohort (33.3%) with Health Survey for England (HSE) 2015 adults aged 16–24 years (39.0%) [31] and a 2021–2023 UK university cohort (32.4%) [32]. External values are contextual benchmarks only and were not statistically compared with the DMU cohort. The DMU and HSE estimates used measured height and weight, whereas the 2021–2023 university estimate was based on self-reported BMI. BIA, bioelectrical impedance analysis; BMI, body mass index; HSE, Health Survey for England; IQR, interquartile range.

The multidimensional adiposity profile is summarised in Figure 2. Although 33.3% of participants had BMI ≥ 25 kg/m^2^, the BIA-derived visceral fat index was concentrated at the lower end of the manufacturer’s rating scale (range 1–9; median 2 [IQR 1–4]). For descriptive context, BMI ≥ 25 kg/m^2^ prevalence was 39.0% among Health Survey for England 2015 participants aged 16–24 years and 32.4% in a 2021–2023 UK university cohort [31,32]. These external estimates were not statistically compared with the present cohort because of differences in sampling, study period and BMI ascertainment.

### 3.4. Secondary and Complementary Body Composition Outcomes

Associations with weight, muscle mass, body water percentage, fat mass and FMI are reported in Table S2 and displayed in Figure S1. No secondary/complementary Model 2 association remained significant after the separate BH-FDR correction.

The smallest nominal *p*-values were observed for Plant-Rich score with muscle mass (β = 0.152, 95% CI −0.011 to 0.314; *p* = 0.067; *q* = 0.510) and Animal-Source Foods with body water percentage (β = 0.147, 95% CI −0.011 to 0.306; *p* = 0.068; *q* = 0.510). These estimates did not meet either conventional two-sided significance after correction or the prespecified multiplicity criterion and were treated as secondary signals rather than independent findings.

### 3.5. Sensitivity, Diagnostic and Exploratory Analyses

When all three dietary pattern scores were entered simultaneously, no association with a primary outcome was statistically significant (Table S3). For the visceral fat index, the mutually adjusted estimates were β = 0.265 for Plant-Rich (*p* = 0.271), β = −0.154 for Animal-Source Foods (*p* = 0.135) and β = 0.037 for Energy-Dense/Discretionary (*p* = 0.818).

Using the standardised log1p-transformed visceral fat index, the single-pattern model for Animal-Source Foods produced β = −0.189 (95% CI −0.377 to −0.0003; *p* = 0.0497), while Plant-Rich and Energy-Dense/Discretionary remained non-significant (Table S4). When all three patterns were included simultaneously in the transformed-outcome model, the Animal-Source estimate attenuated to β = −0.156 (95% CI −0.361 to 0.048; *p* = 0.132). The nominal result in the single-pattern transformed analysis was therefore considered a sensitivity signal and did not alter the primary inference.

Excluding the two participants in the Other geographical background category yielded similar estimates and did not materially change the direction or interpretation of any primary association (Table S5). For example, Animal-Source Foods remained inversely but non-significantly associated with visceral fat index (β = −0.169, 95% CI −0.384 to 0.046; *p* = 0.122). Exploratory effect modification analyses provided no evidence that the primary dietary pattern associations differed across the Africa, Asia and Europe geographical background categories. None of the nine omnibus interaction tests was nominally significant (minimum *p* = 0.475), and none was significant after BH-FDR correction (minimum *q* = 0.944) (Table S9). Geographical background-specific standardised slopes and 95% confidence intervals are also reported in Table S9.

Additional adjustment for standardised MVPA did not materially alter any of the nine primary Model 2 associations (Table S10). The maximum absolute change in the standardised dietary pattern coefficient was 0.010 SD, no coefficient changed direction and there were no changes in nominal *p* < 0.05 or BH-FDR *q* < 0.05 status. Thus, additional adjustment for MVPA did not alter the overall interpretation of the primary dietary pattern–adiposity associations.

Primary model diagnostics showed low multicollinearity, with maximum VIF values below 1.9 across all nine models (Table S6). Breusch–Pagan tests indicated evidence of heteroscedasticity in the three BMI models (*p* = 0.021, 0.044 and 0.020 for Plant-Rich, Animal-Source Foods and Energy-Dense/Discretionary, respectively), supporting the use of HC3 robust inference. Diagnostic criteria identified influential observations in several models. Sensitivity refitting after removal of flagged observations did not generate a consistent alternative pattern of association (Table S7). A nominal Plant-Rich association with body fat percentage was observed after removal of eight flagged observations (β = 0.218, 95% CI 0.023 to 0.413; *p* = 0.029); however, because this estimate arose only after diagnostic exclusions and was not the prespecified primary analysis, it was not interpreted as confirmatory evidence.

In the exploratory logistic regression analyses, Plant-Rich score was associated with an odds ratio of 1.50 (95% CI 0.85–2.63; *p* = 0.158; *q* = 0.237) for BMI ≥ 25 kg/m^2^, Animal-Source Foods with an odds ratio of 0.67 (95% CI 0.42–1.07; *p* = 0.092; *q* = 0.237), and Energy-Dense/Discretionary with an odds ratio of 0.97 (95% CI 0.58–1.63; *p* = 0.906; *q* = 0.906) (Table S8). No model displayed the predefined instability flag, and none provided evidence of an exploratory association after FDR correction.

## 4. Discussion

### 4.1. Principal Findings and Practical Interpretation

In this cohort of 111 university students aged 18–22 years, none of the nine prespecified dietary pattern–adiposity associations was robust after multivariable adjustment and false discovery rate correction. The principal scientific interpretation is that, in the prespecified primary model conditioning on total energy intake, the present data provide no robust evidence that dietary pattern composition was independently associated with contemporary BMI, body fat percentage or BIA-derived visceral fat index. Thus, within the precision available in this cohort, dietary pattern composition did not show a clear independent adiposity association at a given level of overall energy intake [15,16,17,25]. This interpretation was not materially altered by the mutually adjusted, transformed-outcome, geographical background, physical activity-adjusted or influence sensitivity analyses.

This finding should not, however, be interpreted as demonstrating that dietary pattern composition is irrelevant or that the three patterns are equivalent. The fixed analytical sample of 111 participants provided 80% power to detect an incremental effect of approximately *f*^2^ = 0.076 (partial *R*^2^ = 0.071) at the nominal *α* = 0.05 level before multiplicity correction. Smaller independent associations therefore remain compatible with the observed confidence intervals. The data consequently weigh against large or robust independent pattern–adiposity associations in this cohort while retaining uncertainty regarding more modest effects.

This interpretation should also be considered in the context of heterogeneous findings in university and young-adult populations. Previous studies using empirically derived dietary patterns reported associations ranging from more favourable adiposity or body composition profiles for particular patterns to weak or null-adjusted relationships [8,9,33,34]. Such heterogeneity is expected because empirical patterns are population-specific and because studies differ in dietary composition, overall intake, adiposity phenotype and analytical specification.

The role of total energy intake is central to interpreting the present findings. Energy adjustment changes the contrast being estimated: Model 2 addresses the association between dietary pattern composition and adiposity conditional on total energy intake rather than the total effect associated with consuming more of the foods represented by a given pattern [15,16,17,25]. Importantly, none of the nine Model 1 associations was nominally statistically significant before energy adjustment, and addition of total energy intake did not uniformly attenuate the dietary pattern coefficients. The results are nevertheless compatible with the possibility that overall energy consumption captures adiposity-related variation that overlaps with the empirical dietary pattern scores. However, they do not establish that total energy intake is a stronger causal determinant of adiposity than dietary composition, because comparative causal importance was not directly tested and total energy intake was itself estimated from FFQ data. The defensible interpretation is therefore that no clear independent contribution of dietary pattern composition to contemporary adiposity was detectable after conditioning on total energy intake, rather than that dietary composition has no biological relevance.

Contemporary adiposity alone therefore provides an incomplete basis on which to evaluate broader dietary choices. Nutritional quality, environmental sustainability, individual ethical preferences—including considerations related to animal welfare—and evidence concerning longer-term cardiometabolic health may remain relevant when evaluating dietary patterns [12,13,14,35,36]. Importantly, the present Plant-Rich component was an empirical PCA-derived pattern rather than a vegetarian, vegan or predefined healthful plant-based diet, and environmental or ethical outcomes were not measured in this study.

### 4.2. Comparison with Dietary Pattern Studies in University and Young-Adult Populations

The present findings can be contrasted most directly with those of Blondin et al. [8], who examined empirically derived dietary patterns in US university students. Greater adherence to their Prudent pattern was associated with more favourable BMI and body fat outcomes, demonstrating that diet–adiposity relationships can be detectable within university populations. However, the Prudent pattern was not equivalent to the present Plant-Rich component, and the studies differed in sample size, dietary composition, covariate structure and population characteristics. The contrast is therefore more informative as evidence of heterogeneity between young-adult dietary pattern solutions than as evidence of conflicting biological effects.

Similarly, Ul Haq et al. [9] identified Western, meat, and vegetables/fruit patterns among 695 Chinese medical university students and examined a broad set of body composition measures. The Western pattern was positively associated with the FMI/FFMI ratio, while marked sex differences occurred across several body composition characteristics. This study is particularly relevant because it demonstrates the additional information available when dietary pattern analysis is extended beyond BMI to measures of fat and lean mass and visceral fat index.

More recent evidence indicates that weak or null-adjusted associations between empirical dietary patterns and adiposity-related outcomes are not unique to the present cohort. In a 2026 cross-sectional study of 349 Ecuadorian university students aged 18–26 years, Fonseca-Pérez et al. [34] derived dietary dimensions using PCA and subsequently generated mutually exclusive dietary profiles through clustering. After multivariable adjustment, the resulting empirical dietary patterns were not independently associated with excess body weight, relative muscle mass or handgrip strength. The same population nevertheless displayed considerable heterogeneity in body composition and central adiposity. Considered alongside the favourable Prudent pattern associations reported by Blondin et al. and the present findings, these observations suggest that the cross-sectional discriminatory capacity of empirical dietary patterns is not uniform across university populations and may depend on the dietary covariance structure, phenotype assessed and characteristics of the underlying population.

Taken together, variation between studies is expected because empirically derived PCA patterns are population-specific and differ in food composition, covariance structure, sample characteristics and outcome measurement. Similarly labelled patterns should therefore not be assumed to represent equivalent nutritional exposures. The narrow 18–22 years age range of the present cohort additionally provides a focused assessment of emerging adulthood while reducing variation attributable to age.

### 4.3. Cross-Sectional Findings in the Context of Longer-Term Evidence

The cross-sectional findings should be distinguished from evidence concerning longer-term dietary trajectories. Longitudinal studies beginning in early adulthood have reported associations between dietary behaviour and subsequent changes in body weight, adiposity or cardiometabolic outcomes [10,11,12]. These studies provide relevant context for the present results but cannot establish that delayed phenotypic effects occurred in this cohort.

Accordingly, the present analysis addresses contemporaneous associations at the time of assessment. Determining whether sustained dietary behaviours subsequently predict divergence in adiposity requires repeated measurements of both diet and body composition. Cross-sectional and longitudinal evidence should therefore be regarded as addressing complementary rather than interchangeable questions.

### 4.4. Empirical Dietary Patterns Should Not Be Interpreted as A Priori Healthfulness Scores

The direction of several point estimates underscores why empirical dietary pattern labels require careful interpretation. Plant-Rich scores were positively rather than inversely associated with BMI and visceral fat index at the point estimate level, whereas Animal-Source Foods showed negative estimates across the three primary adiposity outcomes. None of these associations was robust after multiplicity correction, and they should not be interpreted as evidence that plant foods increase adiposity or that animal-source foods are protective.

The Plant-Rich component was characterised principally by pulses, nuts/seeds, vegetables, cereals/grains and fruit, with fish/seafood also contributing. It was not designed to reproduce a Mediterranean Diet Score, Eatwell Guide adherence measure or healthful plant-based diet index. Conversely, the Animal-Source Foods pattern combined nutritionally heterogeneous foods including red/processed meat, poultry, fish/seafood, eggs and dairy/alternatives. The Energy-Dense/Discretionary component likewise contained several foods commonly considered discretionary but also cereals/grains and dairy/alternatives.

Prospective evidence supports the distinction between plant predominance and plant food quality. Healthful plant-based dietary indices have generally shown more favourable associations with adiposity than less-healthful plant-based patterns [13,14]. An empirically derived plant-rich PCA component should therefore not be considered equivalent to a healthful plant-based, Mediterranean or other a priori dietary quality score [37]. The present findings concern these specific cohort-derived dietary dimensions and should not be interpreted as contradicting evidence obtained using differently constructed dietary quality indices.

### 4.5. Adiposity Phenotype, Health Risk and the Value of Looking Beyond BMI

The present cohort provides a useful illustration of why population health cannot be inferred from a single anthropometric measure. The BMI distribution was heterogeneous rather than uniformly shifted towards excess weight: 9.0% of participants were underweight, 57.7% were within the healthy weight category and 33.3% had BMI ≥ 25 kg/m^2^, comprising 25.2% who were overweight and 8.1% with obesity. Thus, both low and elevated BMI categories were already represented within this narrow-age population, while approximately one-third showed a BMI-defined excess weight phenotype.

The proportion is also informative when placed cautiously in the UK context. In the 2015 Health Survey for England, approximately 39% of adults aged 16–24 years were classified with BMI ≥ 25 kg/m^2^, based on measured height and weight [31]. A much larger contemporary UK university study conducted during 2021–2023 reported a corresponding prevalence of 32.4% among students with available self-reported BMI [32]. The latter study included 6327 university students overall and therefore provides a valuable contemporary benchmark, although differences in measurement and cohort structure prevent direct statistical comparison.

Against this BMI profile, the visceral fat distribution presents a different picture. Every observed Tanita visceral fat rating was between 1 and 9, whereas the manufacturer’s elevated range begins at 13 [20]. The instrument therefore did not identify an elevated visceral fat rating in any participant. This should not be interpreted as proof of low cardiometabolic risk; the Tanita rating is an indirect BIA-derived estimate and not direct quantification of visceral adipose tissue by MRI or CT. Nevertheless, the contrast with BMI is informative because BMI-defined weight status and the BIA-derived visceral fat index did not reflect the same degree or distribution of adiposity burden within this young population.

Evidence from other student populations supports the importance of this multidimensional assessment. Liu et al. recently reported hidden obesity—excess body fat despite normal BMI—in 17.0% of 993 Chinese university students, with particularly marked sex differences [38]. While those findings cannot be transferred quantitatively to the present population, they reinforce the principle that BMI alone may both over- and under-characterise aspects of body composition.

Taken together, these findings support a multidimensional rather than binary interpretation of adiposity in emerging adulthood. The coexistence of BMI-defined excess weight with comparatively restricted BIA-derived visceral fat values illustrates that different adiposity indicators capture related but non-equivalent phenotypic information. This does not classify the cohort globally as either healthy or at risk; rather, it reinforces the value of combining anthropometric and body composition measures when characterising young-adult adiposity.

### 4.6. Strengths and Interpretive Limitations

Several features strengthen the present analysis: The narrow 18–22-year age range provides a focused assessment of emerging adulthood; height and weight were measured rather than self-reported; adiposity was characterised across multiple complementary phenotypes; and the dietary pattern scores were established independently of the adiposity analyses. The inferential framework further limited selective interpretation through prespecified covariate structures, HC3 robust standard errors, a defined nine-association multiplicity family, BH-FDR correction and complementary sensitivity and influence analyses. Geographical background was explicitly included in the primary adjustment structure, and additional interaction analyses provided no evidence of effect modification across the three main geographical background categories.

The principal limitations concern precision, exposure and outcome measurement and residual confounding. The fixed analytical sample of 111 participants limits precision for small associations and subgroup analyses, while continent of birth provides only a broad descriptor of geographical background rather than genetic ancestry or ethnicity. Dietary and energy intakes were estimated using an FFQ without concurrent reference dietary assessment in this cohort and are therefore subject to measurement and reporting error. FFQs are well suited to characterising habitual dietary intake and ranking individuals, but estimates of absolute intake, including total energy intake, require greater caution [39]. In an EPIC validation study comparing questionnaire-derived energy intake with total energy expenditure measured by doubly labelled water, energy intake and expenditure were only moderately correlated and questionnaire-derived energy intake was underreported by approximately 22% on average [40]. Consequently, total energy intake in the present study should be regarded as an estimated covariate rather than an error-free measure, adding uncertainty to the interpretation of associations conditioned on energy intake. Physical activity was assessed in the parent study and was incorporated here only as an additional sensitivity covariate because the present analysis was specifically designed to examine dietary pattern–adiposity associations. A detailed evaluation of physical activity intensity, sedentary behaviour and their relationships with body composition was therefore beyond the scope of the present study. Body composition was assessed using BIA rather than a criterion method, although standardised preassessment conditions were used and BMI was independently derived from measured height and weight. Finally, the cross-sectional and historical design limits causal and current prevalence inference. These considerations primarily constrain precision, causal interpretation and generalisability rather than the internal consistency of the prespecified within-cohort analyses.

### 4.7. Future Research Priorities

Future studies should prioritise larger prospective and multi-university cohorts with repeated assessment of dietary patterns and body composition throughout emerging adulthood. Repeated dietary assessment should ideally be accompanied by repeated and detailed assessment of physical activity, sedentary behaviour and other relevant lifestyle factors, DXA or imaging-based adiposity assessment where feasible and cardiometabolic biomarkers. Such designs would permit more precise evaluation of effect modification and determine whether dietary pattern differences that are limited for contemporaneous adiposity predict subsequent divergence in body composition or metabolic health.

## 5. Conclusions

In this geographically diverse cohort of university students aged 18–22 years, no robust independent association was detected between the three empirically derived whole-diet pattern scores and contemporary BMI, body fat percentage or BIA-derived visceral fat index after conditioning on total energy intake and applying false discovery rate correction. The findings therefore suggest limited detectable independent relevance of dietary pattern composition for contemporary adiposity in this cohort. However, given the precision available with *N* = 111, smaller independent associations cannot be excluded.

Importantly, none of the corresponding Model 1 associations was nominally statistically significant before energy adjustment, and inclusion of total energy intake did not uniformly attenuate the dietary pattern coefficients. The results are therefore compatible with the possibility that overall energy intake captures adiposity-related variation that overlaps with dietary pattern composition, but they do not establish that energy intake is a stronger causal determinant of adiposity. Additional adjustment for moderate-to-vigorous physical activity did not materially alter the overall interpretation.

These findings concern contemporaneous adiposity rather than the broader nutritional or long-term health consequences of dietary behaviour. Larger prospective studies with repeated assessment of diet, total energy intake and body composition are required to determine whether dietary patterns established during emerging adulthood predict subsequent divergence in adiposity and cardiometabolic health.

## Figures and Tables

**Table 1:** Characteristics of the study population and adiposity/body composition profile.

Characteristic	Unit	Mean ± SD or *n* (%)	Median [IQR]
Participants	*N*	111	—
Age	years	20.4 ± 1.1	20 [20,21]
Sex: Female	*n* (%)	78 (70.3%)	—
Sex: Male	*n* (%)	33 (29.7%)	—
Geographical background: Africa	*n* (%)	41 (36.9%)	—
Geographical background: Asia	*n* (%)	41 (36.9%)	—
Geographical background: Europe	*n* (%)	27 (24.3%)	—
Geographical background: Other	*n* (%)	2 (1.8%)	—
Total energy intake	kcal/day	2524 ± 1256	2245 [1664–3115]
Height	cm	167.2 ± 9.3	167.0 [160.1–173.3]
Weight	kg	66.4 ± 13.5	64.3 [57.4–75.7]
BMI	kg/m^2^	23.69 ± 4.09	23.35 [21.19–25.98]
Body fat	%	25.7 ± 10.4	24.8 [18.7–31.7]
Visceral fat index	index	2.7 ± 2.0	2.0 [1.0–4.0]
Muscle mass	kg	46.4 ± 8.8	44.0 [39.9–51.9]
Body water	%	53.0 ± 6.7	52.9 [48.9–57.3]
Fat mass	kg	17.7 ± 9.5	16.1 [10.9–21.5]
Fat mass index	kg/m^2^	6.39 ± 3.48	5.95 [4.08–7.99]
Basal metabolic rate	kcal/day	1514 ± 256	1454 [1320–1673]
BMI category: underweight	*n* (%)	10 (9.0%)	—
BMI category: healthy weight	*n* (%)	64 (57.7%)	—
BMI category: overweight	*n* (%)	28 (25.2%)	—
BMI category: obesity	*n* (%)	9 (8.1%)	—

Values are reported for the complete analytical cohort (*N* = 111). BMI categories were defined using conventional adult cut points: underweight, <18.5 kg/m^2^; healthy weight, 18.5–24.9 kg/m^2^; overweight, 25.0–29.9 kg/m^2^; and obese, ≥30.0 kg/m^2^. Visceral fat index is a bioelectrical impedance-derived estimate from the Tanita SC-330-S and does not represent direct anatomical quantification of visceral adipose tissue. BMI, body mass index; IQR, interquartile range; SD, standard deviation.

**Table 2:** Associations of the three pre-established dietary pattern scores with the prespecified primary adiposity outcomes.

Outcome	Dietary Pattern	Model 1: β (95% CI)	*p*	Model 2: β (95% CI)	*p*	BH-FDR *q*
BMI	Plant-Rich	+0.156 (−0.054 to +0.366)	0.144	+0.242 (−0.142 to +0.627)	0.214	0.481
	Animal-Source Foods	−0.113 (−0.325 to +0.100)	0.297	−0.122 (−0.343 to +0.099)	0.276	0.496
	Energy-Dense/Discretionary	−0.012 (−0.287 to +0.263)	0.929	−0.017 (−0.305 to +0.271)	0.908	0.908
Body fat (%)	Plant-Rich	+0.149 (−0.019 to +0.317)	0.081	+0.125 (−0.146 to +0.397)	0.362	0.543
	Animal-Source Foods	−0.061 (−0.219 to +0.096)	0.441	−0.104 (−0.257 to +0.049)	0.181	0.481
	Energy-Dense/Discretionary	+0.080 (−0.127 to +0.286)	0.447	+0.024 (−0.225 to +0.272)	0.851	0.908
Visceral fat index	Plant-Rich	+0.159 (−0.058 to +0.377)	0.150	+0.250 (−0.143 to +0.644)	0.210	0.481
	Animal-Source Foods	−0.159 (−0.345 to +0.027)	0.092	−0.172 (−0.358 to +0.014)	0.069	0.481
	Energy-Dense/Discretionary	−0.035 (−0.298 to +0.228)	0.792	−0.050 (−0.332 to +0.231)	0.722	0.908

β values are standardised regression coefficients. Model 1 adjusted for age, sex and geographical background. Model 2, the prespecified primary model, additionally adjusted for log-transformed total energy intake. HC3 robust standard errors were used for inference. Benjamini–Hochberg false discovery rate (BH-FDR) correction was applied jointly to the nine Model 2 *p*-values (3 dietary patterns × 3 primary outcomes). No primary association met *q* < 0.05. CI, confidence interval.

## Data Availability

Derived, non-identifiable analytical outputs, plotting datasets and R code supporting the findings of this study are publicly available on Figshare at https://doi.org/10.6084/m9.figshare.33792070. The participant-level dataset is not publicly available because it contains human participant information subject to ethical, institutional and data protection requirements. Requests for additional de-identified participant-level data may be considered by the corresponding author in accordance with the applicable ethical and institutional conditions.

## Supplementary Materials

The following supporting information can be downloaded at https://www.mdpi.com/article/10.3390/nu18203320/s1: Figure S1: Associations of the three validated dietary-pattern scores with secondary and complementary body-composition outcomes in Model 2. Table S1: Principal food-group loadings and variance explained for the three validated dietary patterns. Table S2: Associations of dietary-pattern scores with secondary and complementary body-composition outcomes. Table S3: Sensitivity analyses with all three dietary-pattern scores entered simultaneously. Table S4: Sensitivity analysis using log1p-transformed visceral-fat index. Table S5: Sensitivity analysis excluding the geographical-background category Other (*n* = 2). Table S6: Diagnostic summary for the nine prespecified primary Model 2 linear regressions. Table S7: Influence-sensitivity refits for the nine prespecified primary Model 2 associations. Table S8: Exploratory logistic-regression associations with BMI ≥ 25 kg/m^2^ versus <25 kg/m^2^. Table S9: Exploratory effect-modification analyses of the nine primary dietary-pattern–adiposity associations across geographical-background categories. Table S10: Physical-activity-adjusted sensitivity analyses of the nine primary dietary-pattern–adiposity associations.
