# Supplementary material for: Empirically Derived Whole-Diet Patterns and Multidimensional Adiposity in Emerging Adulthood: A Cross-Sectional Study of Geographically Diverse Students at a UK University
Source: Nutrients. 2026 Oct 10;18(20):3320. doi: 10.3390/nu18203320 (PMC13708284; doi:10.3390/nu18203320)
Supplement: Supplementary file 1 [file nutrients-18-03320-s001.zip › Supplementary Figure S1.pdf]

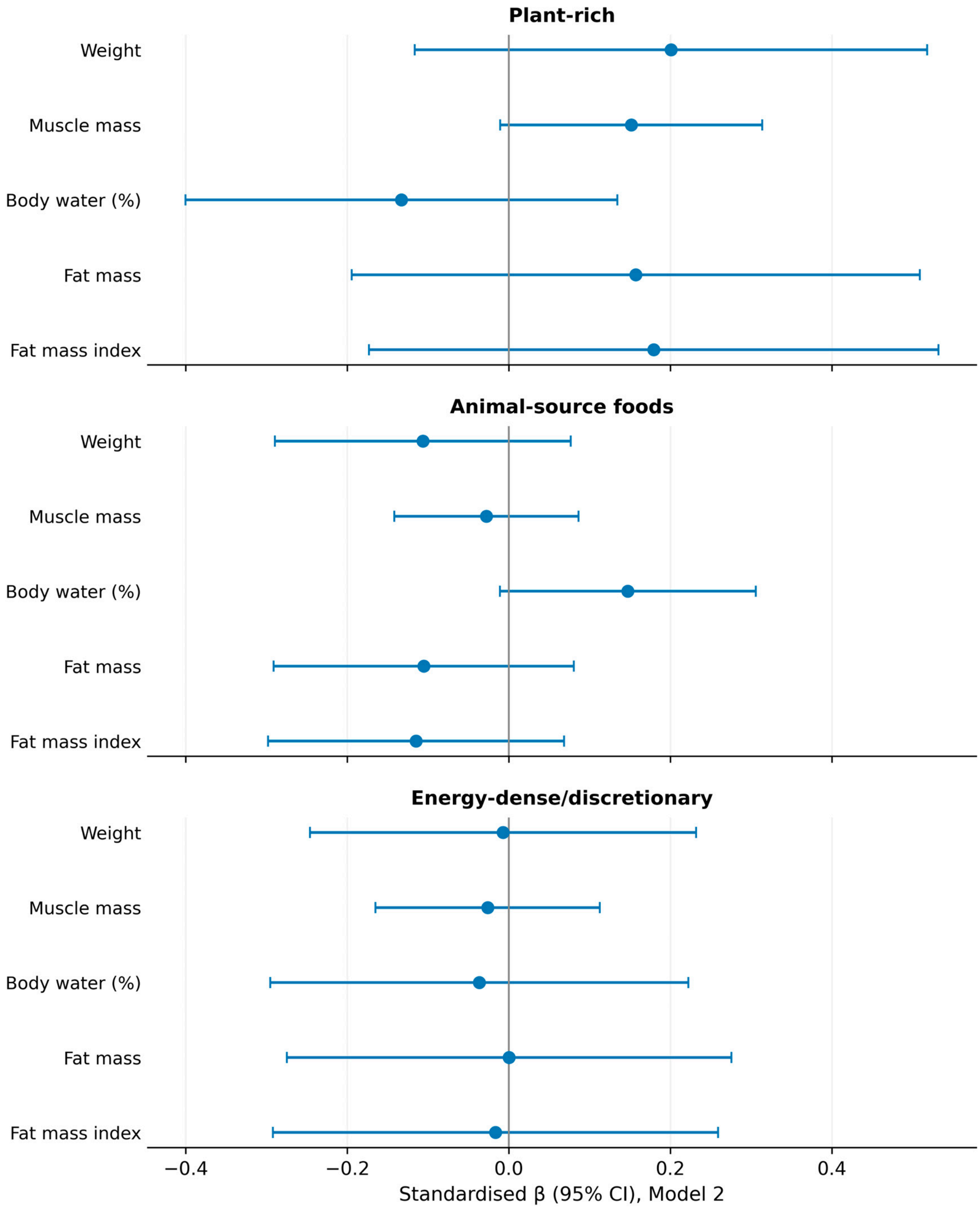

Figure S1. Associations of the three validated dietary-pattern scores with secondary and complementary body-composition outcomes in Model 2.

Points represent standardised β coefficients and horizontal bars represent 95% confidence intervals. Models were adjusted for age, sex, geographical background and log-transformed total energy intake; models for weight, muscle mass and fat mass were additionally adjusted for height. HC3 robust standard errors were used for inference. The vertical line indicates β = 0. CI, confidence interval.
