# Supplementary material for: Empirically Derived Whole-Diet Patterns and Multidimensional Adiposity in Emerging Adulthood: A Cross-Sectional Study of Geographically Diverse Students at a UK University
Source: Nutrients. 2026 Oct 10;18(20):3320. doi: 10.3390/nu18203320 (PMC13708284; doi:10.3390/nu18203320)
Supplement: Supplementary file 1 [file nutrients-18-03320-s001.zip › Supplementary Tables S1–S10.pdf]

**Table S1.** Principal food-group loadings and variance explained for the three validated dietary patterns.

| Dietary pattern | Principal positive food-group loadings | Variance explained (%) |
|---|---|---|
| Plant-rich | Pulses (0.790); Nuts/Seeds (0.670); Vegetables (0.661); Cereals/Grains (0.619); Fruit (0.539); Fish/Seafood (0.341) | 16.30 |
| Animal-source foods | Red/processed meat (0.766); Poultry (0.765); Fish/Seafood (0.649); Eggs (0.531); Dairy/Alternatives (0.380) | 15.18 |
| Energy-dense/discretionary | Fats/Oils (0.788); Discretionary foods (0.648); Soft/energy/sports drinks (0.551); Alcoholic beverages (0.480); Cereals/Grains (0.450); Dairy/Alternatives (0.448) | 14.76 |

Values in parentheses are varimax-rotated PCA loadings from the previously derived and validated three-component dietary-pattern solution. The three patterns jointly explained 46.25% of the variance in food-group intake. Pattern labels are descriptive empirical labels and should not be interpreted as prespecified healthfulness scores. Participant-level pattern scores were derived before the present adiposity analyses and were not recalculated.

**Table S2.** Associations of dietary-pattern scores with secondary and complementary body-composition outcomes.

| Outcome | Dietary pattern | Model 1: β (95% CI) | *p* | Model 2: β (95% CI) | *p* | BH-FDR *q* |
|---|---|---|---|---|---|---|
| Weight | Plant-rich | +0.119 (-0.069 to +0.307) | 0.212 | +0.201 (-0.116 to +0.518) | 0.212 | 0.612 |
| | Animal-source foods | -0.103 (-0.280 to +0.074) | 0.253 | -0.106 (-0.289 to +0.077) | 0.252 | 0.612 |
| | Energy-dense/discretionary | -0.017 (-0.244 to +0.209) | 0.879 | -0.007 (-0.246 to +0.232) | 0.952 | 0.998 |
| Muscle mass | Plant-rich | +0.064 (-0.059 to +0.187) | 0.305 | +0.152 (-0.011 to +0.314) | 0.067 | 0.510 |
| | Animal-source foods | -0.044 (-0.148 to +0.059) | 0.396 | -0.028 (-0.142 to +0.086) | 0.629 | 0.943 |
| | Energy-dense/discretionary | -0.050 (-0.166 to +0.066) | 0.390 | -0.026 (-0.165 to +0.113) | 0.710 | 0.968 |
| Body water (%) | Plant-rich | -0.159 (-0.321 to +0.003) | 0.055 | -0.133 (-0.400 to +0.134) | 0.326 | 0.612 |
| | Animal-source foods | +0.099 (-0.065 to +0.262) | 0.236 | +0.147 (-0.011 to +0.306) | 0.068 | 0.510 |
| | Energy-dense/discretionary | -0.093 (-0.306 to +0.121) | 0.391 | -0.037 (-0.295 to +0.222) | 0.780 | 0.975 |
| Fat mass | Plant-rich | +0.116 (-0.089 to +0.320) | 0.264 | +0.157 (-0.195 to +0.509) | 0.378 | 0.629 |
| | Animal-source foods | -0.088 (-0.271 to +0.095) | 0.344 | -0.105 (-0.291 to +0.081) | 0.264 | 0.612 |
| | Energy-dense/discretionary | +0.012 (-0.238 to +0.261) | 0.927 | +0.000 (-0.275 to +0.276) | 0.998 | 0.998 |
| Fat mass index | Plant-rich | +0.147 (-0.042 to +0.336) | 0.127 | +0.179 (-0.173 to +0.532) | 0.315 | 0.612 |
| | Animal-source foods | -0.090 (-0.272 to +0.092) | 0.330 | -0.115 (-0.298 to +0.068) | 0.217 | 0.612 |
| | Energy-dense/discretionary | +0.019 (-0.236 to +0.273) | 0.886 | -0.017 (-0.292 to +0.259) | 0.905 | 0.998 |

β values are standardised coefficients. Model 1 adjusted for age, sex and geographical background; Model 2 additionally adjusted for log-transformed total energy intake. Weight, muscle mass and fat mass models additionally included height. HC3 robust standard errors were used. BH-FDR q-values refer to the prespecified Model 2 secondary/complementary family and are separate from the nine-test primary family. FMI, fat mass index.

**Table S3.** Sensitivity analyses with all three dietary-pattern scores entered simultaneously.

| Outcome | Role | Dietary pattern | β (95% CI) | *p* | Height adjusted |
|---|---|---|---|---|---|
| BMI | Primary | Plant-rich | +0.308 (-0.152 to +0.768) | 0.187 | No |
| | | Animal-source foods | -0.081 (-0.351 to +0.189) | 0.552 | No |
| | | Energy-dense/discretionary | +0.118 (-0.236 to +0.472) | 0.510 | No |
| Body fat (%) | Primary | Plant-rich | +0.175 (-0.129 to +0.479) | 0.256 | No |
| | | Animal-source foods | -0.074 (-0.244 to +0.096) | 0.388 | No |
| | | Energy-dense/discretionary | +0.091 (-0.189 to +0.370) | 0.521 | No |
| Visceral-fat index | Primary | Plant-rich | +0.265 (-0.210 to +0.741) | 0.271 | No |
| | | Animal-source foods | -0.154 (-0.357 to +0.049) | 0.135 | No |
| | | Energy-dense/discretionary | +0.037 (-0.283 to +0.357) | 0.818 | No |
| Weight | Secondary | Plant-rich | +0.257 (-0.121 to +0.636) | 0.181 | Yes |
| | | Animal-source foods | -0.080 (-0.308 to +0.148) | 0.488 | Yes |
| | | Energy-dense/discretionary | +0.095 (-0.204 to +0.393) | 0.531 | Yes |
| Muscle mass | Secondary | Plant-rich | +0.192 (-0.030 to +0.414) | 0.089 | Yes |
| | | Animal-source foods | -0.009 (-0.147 to +0.129) | 0.898 | Yes |
| | | Energy-dense/discretionary | +0.069 (-0.137 to +0.275) | 0.511 | Yes |
| Body water (%) | Complementary | Plant-rich | -0.182 (-0.485 to +0.120) | 0.235 | No |
| | | Animal-source foods | +0.117 (-0.056 to +0.290) | 0.184 | No |
| | | Energy-dense/discretionary | -0.093 (-0.393 to +0.207) | 0.540 | No |
| Fat mass | Secondary-derived | Plant-rich | +0.200 (-0.194 to +0.594) | 0.317 | Yes |
| | | Animal-source foods | -0.086 (-0.308 to +0.136) | 0.445 | Yes |
| | | Energy-dense/discretionary | +0.071 (-0.234 to +0.375) | 0.646 | Yes |
| Fat mass index | Secondary-derived | Plant-rich | +0.214 (-0.189 to +0.616) | 0.294 | No |
| | | Animal-source foods | -0.091 (-0.304 to +0.122) | 0.399 | No |
| | | Energy-dense/discretionary | +0.065 (-0.241 to +0.372) | 0.674 | No |

All models included the three dietary-pattern scores simultaneously and adjusted for age, sex, geographical background and log-transformed total energy intake. Height was additionally included for weight, muscle mass and fat mass. These mutually adjusted models were prespecified sensitivity analyses and did not replace the single-pattern primary models.

**Table S4.** Sensitivity analysis using log1p-transformed visceral-fat index.

| **Model specification** | **Dietary pattern** | **β (95% CI)** | ***p*** |
|---|---|---|---|
| Single pattern | Plant-rich | +0.226 (-0.115 to +0.567) | 0.191 |
| | Animal-source foods | -0.189 (-0.377 to -0.000) | 0.050 |
| | Energy-dense/discretionary | -0.001 (-0.275 to +0.274) | 0.996 |
| All patterns mutually adjusted | Plant-rich | +0.272 (-0.146 to +0.689) | 0.199 |
| | Animal-source foods | -0.156 (-0.361 to +0.048) | 0.132 |
| | Energy-dense/discretionary | +0.090 (-0.232 to +0.412) | 0.582 |

The outcome was the standardised log1p-transformed visceral-fat index. Models were adjusted for age, sex, geographical background and log-transformed total energy intake. These analyses were prespecified sensitivity analyses and were not included in the nine-test primary multiplicity family.

**Table S5.** Sensitivity analysis excluding the geographical-background category Other (*n* = 2).

| Outcome | Dietary pattern | *n* | β (95% CI) | *p* |
|---|---|---|---|---|
| BMI | Plant-rich | 109 | +0.258 (-0.133 to +0.649) | 0.194 |
| | Animal-source foods | 109 | -0.164 (-0.370 to +0.042) | 0.117 |
| | Energy-dense/discretionary | 109 | -0.025 (-0.319 to +0.268) | 0.864 |
| Body fat (%) | Plant-rich | 109 | +0.128 (-0.151 to +0.406) | 0.365 |
| | Animal-source foods | 109 | -0.118 (-0.286 to +0.050) | 0.166 |
| | Energy-dense/discretionary | 109 | +0.024 (-0.230 to +0.278) | 0.850 |
| Visceral-fat index | Plant-rich | 109 | +0.244 (-0.159 to +0.647) | 0.233 |
| | Animal-source foods | 109 | -0.169 (-0.384 to +0.046) | 0.122 |
| | Energy-dense/discretionary | 109 | -0.042 (-0.329 to +0.246) | 0.774 |

These models reproduced the primary Model 2 specification after excluding the two participants classified as Other for geographical background. The direction and interpretation of the primary findings were not materially altered.

**Table S6.** Diagnostic summary for the nine prespecified primary Model 2 linear regressions.

| Outcome | Pattern | BP $p$ | Max VIF | Max Cook's D | $n$ Cook's D > 4/n | Max leverage | $n$ leverage > 2p/n | Max \|studentised residual\| | $n$ \|residual\| >3 | Max \|DFBETA\| exposure | $n$ \|DFBETA\| > 2/√n |
|---|---|---|---|---|---|---|---|---|---|---|---|
| BMI | Plant-rich | 0.021 | 1.83 | 0.221 | 7 | 0.524 | 3 | 3.78 | 3 | 0.92 | 6 |
| | Animal-source foods | 0.044 | 1.85 | 0.182 | 8 | 0.575 | 6 | 4.05 | 3 | 0.56 | 5 |
| | Energy-dense/discretionary | 0.020 | 1.87 | 0.261 | 8 | 0.521 | 3 | 4.11 | 2 | 0.79 | 4 |
| Body fat (%) | Plant-rich | 0.198 | 1.83 | 0.120 | 5 | 0.524 | 3 | 3.24 | 1 | 0.57 | 9 |
| | Animal-source foods | 0.241 | 1.85 | 0.087 | 4 | 0.575 | 6 | 3.00 | 0 | 0.51 | 2 |
| | Energy-dense/discretionary | 0.099 | 1.87 | 0.132 | 5 | 0.521 | 3 | 2.98 | 0 | 0.69 | 11 |
| Visceral-fat index | Plant-rich | 0.156 | 1.83 | 0.206 | 6 | 0.524 | 3 | 3.87 | 2 | 1.01 | 7 |
| | Animal-source foods | 0.180 | 1.85 | 0.136 | 7 | 0.575 | 6 | 3.42 | 3 | 0.47 | 5 |
| | Energy-dense/discretionary | 0.122 | 1.87 | 0.187 | 8 | 0.521 | 3 | 3.40 | 3 | 0.66 | 7 |

BP, Breusch–Pagan test for heteroscedasticity; VIF, variance inflation factor. Influence flags were defined as Cook's D > 4/$n$, leverage > 2$p$/$n$, |studentised residual| > 3, or |DFBETA| > 2/√$n$, where $n$ denotes model sample size and $p$ the number of estimated model parameters. Diagnostic flags were used to identify observations for sensitivity refitting and did not result in automatic exclusion from the primary analyses.

**Table S7.** Influence-sensitivity refits for the nine prespecified primary Model 2 associations.

| Outcome | Pattern | *n* removed | Removed participant IDs | β original | β refit | Δβ | Refit 95% CI | *p* refit |
|---|---|---|---|---|---|---|---|---|
| BMI | Plant-rich | 8 | 4;6;39;41;59;67;73;104 | +0.242 | +0.144 | -0.099 | (-0.064 to +0.351) | 0.173 |
| | Animal-source foods | 11 | 4;6;34;39;41;57;59;67;73;104;110 | -0.122 | -0.068 | +0.054 | (-0.313 to +0.176) | 0.582 |
| | Energy-dense/discretionary | 9 | 4;6;39;41;59;67;73;104;109 | -0.017 | +0.074 | +0.090 | (-0.109 to +0.256) | 0.425 |
| Body fat (%) | Plant-rich | 8 | 4;6;41;42;55;59;67;104 | +0.125 | +0.218 | +0.093 | (+0.023 to +0.413) | 0.029 |
| | Animal-source foods | 9 | 6;34;41;55;57;59;67;104;110 | -0.104 | +0.020 | +0.124 | (-0.230 to +0.270) | 0.872 |
| | Energy-dense/discretionary | 8 | 6;26;41;42;55;59;67;104 | +0.024 | +0.046 | +0.022 | (-0.147 to +0.240) | 0.637 |
| Visceral-fat index | Plant-rich | 9 | 4;6;41;55;59;67;95;104;109 | +0.250 | +0.218 | -0.032 | (-0.002 to +0.438) | 0.052 |
| | Animal-source foods | 12 | 4;6;34;41;57;59;67;95;101;104;109;110 | -0.172 | -0.136 | +0.036 | (-0.445 to +0.173) | 0.384 |
| | Energy-dense/discretionary | 9 | 4;6;41;55;59;67;95;104;109 | -0.050 | +0.052 | +0.103 | (-0.142 to +0.247) | 0.596 |

Participants were excluded only within the influence-refit sensitivity analyses according to the prespecified diagnostic criteria. These refits were used to assess robustness and did not replace the primary Model 2 analyses.

**Table S8.** Exploratory logistic-regression associations with BMI ≥25 kg/m² versus <25 kg/m².

| Dietary pattern | Odds ratio | 95% CI | *p* | BH-FDR *q* | Events/non-events | Instability flag |
|---|---|---|---|---|---|---|
| Plant-rich | 1.50 | 0.85 to 2.63 | 0.158 | 0.237 | 37/74 | No |
| Animal-source foods | 0.67 | 0.42 to 1.07 | 0.092 | 0.237 | 37/74 | No |
| Energy-dense/discretionary | 0.97 | 0.58 to 1.63 | 0.906 | 0.906 | 37/74 | No |

The exploratory outcome comprised 37 participants with BMI ≥25 kg/m² and 74 participants with BMI <25 kg/m². Models used standard logistic regression and adjusted for age, sex, geographical background and log-transformed total energy intake. BH-FDR correction was applied within this three-test exploratory family. These analyses were exploratory and were not part of the confirmatory primary family.

**Table S9.** Exploratory effect-modification analyses of the nine primary dietary-pattern–adiposity associations across geographical-background categories.

| Outcome | Dietary pattern | Europe $\beta$ (95% CI) | Africa $\beta$ (95% CI) | Asia $\beta$ (95% CI) | Omnibus $F$(2,100) | $p$ | BH-FDR $q$ |
|---|---|---|---|---|---|---|---|
| BMI | Plant-rich | +0.133 (-0.504 to +0.769) | +0.264 (-0.081 to +0.610) | +0.437 (-0.110 to +0.983) | 0.412 | 0.663 | 0.944 |
| | Animal-source foods | +0.110 (-0.536 to +0.756) | +0.001 (-0.345 to +0.347) | -0.253 (-0.515 to +0.010) | 0.750 | 0.475 | 0.944 |
| | Energy-dense/discretionary | -0.212 (-1.100 to +0.676) | +0.024 (-0.176 to +0.224) | +0.008 (-0.595 to +0.611) | 0.129 | 0.879 | 0.944 |
| Body fat (%) | Plant-rich | +0.057 (-0.292 to +0.406) | +0.119 (-0.237 to +0.474) | +0.248 (-0.221 to +0.717) | 0.323 | 0.725 | 0.944 |
| | Animal-source foods | -0.036 (-0.563 to +0.492) | -0.118 (-0.532 to +0.295) | -0.132 (-0.340 to +0.075) | 0.057 | 0.944 | 0.944 |
| | Energy-dense/discretionary | -0.172 (-0.665 to +0.321) | +0.017 (-0.245 to +0.280) | +0.117 (-0.357 to +0.591) | 0.378 | 0.686 | 0.944 |
| Visceral-fat index | Plant-rich | +0.112 (-0.557 to +0.781) | +0.242 (-0.170 to +0.654) | +0.445 (-0.026 to +0.916) | 0.637 | 0.531 | 0.944 |
| | Animal-source foods | +0.020 (-0.843 to +0.882) | -0.090 (-0.549 to +0.369) | -0.221 (-0.474 to +0.031) | 0.202 | 0.817 | 0.944 |
| | Energy-dense/discretionary | -0.236 (-1.147 to +0.675) | +0.025 (-0.242 to +0.293) | -0.022 (-0.554 to +0.510) | 0.155 | 0.857 | 0.944 |

Analyses excluded the two participants classified as Other, leaving $N = 109$ (Africa $n = 41$, Asia $n = 41$, Europe $n = 27$). Each model extended the fully adjusted primary Model 2 with a dietary-pattern score × geographical-background interaction while retaining age, sex, geographical background and log-transformed total energy intake. The same outcome and dietary-pattern standardisation and HC3 robust inference used in the primary analyses were retained. Interaction terms were evaluated jointly using an omnibus robust Wald $F$ test; BH-FDR correction was applied separately across the nine interaction tests. Group-specific values are standardised dietary-pattern slopes ($\beta$) with 95% confidence intervals. Geographical background was derived from continent of birth and should not be interpreted as genetic ancestry, ethnicity or cultural identity. No interaction test was nominally significant (minimum $p = 0.475$), and none was significant after BH-FDR correction (minimum $q = 0.944$).

**Table S10.** Physical-activity-adjusted sensitivity analyses of the nine primary dietary-pattern–adiposity associations.

| Outcome | Dietary pattern | *n* | Standardised β (95% CI) | *p* | BH-FDR q |
|---|---|---|---|---|---|
| **BMI** | Plant-rich | 111 | +0.252 (−0.131 to +0.636) | 0.194 | 0.438 |
| | Animal-source foods | 111 | −0.120 (−0.340 to +0.100) | 0.283 | 0.509 |
| | Energy-dense/discretionary | 111 | −0.022 (−0.301 to +0.256) | 0.873 | 0.873 |
| **Body fat (%)** | Plant-rich | 111 | +0.128 (−0.143 to +0.400) | 0.352 | 0.527 |
| | Animal-source foods | 111 | −0.105 (−0.258 to +0.047) | 0.175 | 0.438 |
| | Energy-dense/discretionary | 111 | +0.024 (−0.223 to +0.270) | 0.851 | 0.873 |
| **Visceral-fat index** | Plant-rich | 111 | +0.257 (−0.134 to +0.648) | 0.195 | 0.438 |
| | Animal-source foods | 111 | −0.173 (−0.360 to +0.014) | 0.069 | 0.438 |
| | Energy-dense/discretionary | 111 | −0.054 (−0.327 to +0.220) | 0.698 | 0.873 |

Values are standardised dietary-pattern coefficients (β) with 95% confidence intervals. Each model reproduced the primary fully adjusted Model 2 specification with standardised moderate-to-vigorous physical activity (MVPA) included as an additional covariate; models therefore adjusted for age, sex, geographical background, log-transformed total energy intake and standardised MVPA. The same exposure and outcome standardisation and HC3 robust inference used in the primary analyses were retained. BH-FDR correction was applied separately across the nine dietary-pattern coefficients from this sensitivity analysis. The maximum absolute change relative to the corresponding primary Model 2 coefficient was 0.010 SD; no coefficient changed direction, and no association changed nominal $p < 0.05$ or BH-FDR $q < 0.05$ status. This sensitivity analysis did not replace or modify the prespecified primary Model 2 analyses. CI, confidence interval; MVPA, moderate-to-vigorous physical activity.
